# Kinetics Study of PVA Polymer by Model-Free and Model-Fitting Methods Using TGA

**DOI:** 10.3390/polym16050629

**Published:** 2024-02-26

**Authors:** Zaid Abdulhamid Alhulaybi, Ibrahim Dubdub

**Affiliations:** Chemical Engineering Department, King Faisal University, P.O. Box 380, Al-Ahsa 31982, Saudi Arabia

**Keywords:** polyvinyl alcohol (PVA), decomposition, TGA, kinetics, model-free, model-fitting

## Abstract

Thermogravimetric Analysis (TGA) serves a pivotal technique for evaluating the thermal behavior of Polyvinyl alcohol (PVA), a polymer extensively utilized in the production of fibers, films, and membranes. This paper targets the kinetics of PVA thermal degradation using high three heating rate range 20, 30, and 40 K min^−1^. The kinetic study was performed using six model-free methods: Freidman (FR), Flynn-Wall-Qzawa (FWO), Kissinger-Akahira-Sunose (KAS), Starink (STK), Kissinger (K), and Vyazovkin (VY) for the determination of the activation energy (*E_a_*). TGA showed two reaction stages: the main one at 550–750 K and the second with 700–810 K. But only the first step has been considered in calculating *E_a_*. The average activation energy values for the conversion range (0.1–0.7) are between minimum 104 kJ mol^−1^ by VY to maximum 199 kJ mol^−1^ by FR. Model-fitting has been applied by combing Coats–Redfern (CR) with the master plot (Criado’s) to identify the most convenient reaction mechanism. *E_a_* values gained by the above six models were very similar with the average value of (126 kJ mol^−1^) by CR. The reaction order models-Second order (F2) was recommended as the best mechanism reaction for PVA pyrolysis. Mechanisms were confirmed by the compensation effect. Finally, (∆H, ∆G, and ∆S) parameters were presented and proved that the reaction is endothermic.

## 1. Introduction

Polyvinyl alcohol (PVA) is a versatile synthetic polymer with a wide range of applications in various industries. It is a water-soluble, odorless, and colorless polymer that is known for its biocompatibility, high tensile strength, and excellent film-forming properties [1,2]. The above-mentioned properties make PVA a valuable material for a variety of uses. For instance, in the textile industry, PVA is used as a sizing agent to improve the strength and smoothness of yarns and fabrics [3]. It is also used as a thickener in printing pastes, a binder in non-woven fabrics, and a coating for textiles to enhance their water resistance and stain resistance [4]. PVA’s ability to adhere to fibers and form a smooth film makes it an ideal material for textile applications [5]. PVA is widely used in food packaging due to its excellent barrier properties. It can protect food from moisture, oxygen, and other gases, thereby extending shelf life and preventing spoilage [6]. In addition, PVA films are also transparent, allowing consumers to see the food without compromising its integrity. Additionally, PVA is non-toxic and biodegradable, making it an environmentally friendly choice for food packaging [7]. PVA is used in a variety of medical and pharmaceutical applications due to its biocompatibility and ability to form hydrogels. Hydrogels are water-based gels that can be used to deliver drugs, wound dressings, and other medical products [8]. PVA is also used in the production of contact lenses, artificial skin, and surgical sutures [9]. PVA is used in a variety of construction and building materials due to its adhesive properties and ability to form films [10]. It is used as a binder in plaster, adhesives for drywall and wallpaper, and as a coating for concrete and other surfaces [11]. In addition to the applications mentioned above, PVA is used in a variety of other products, including detergents and soaps, cosmetics and personal care products, adhesives and sealants, paints and coating, toys and games and novel materials and composites [12,13,14].

Given PVA’s favorable attributes, including water solubility, dopant-dependent properties, electrical and optical conductivity, thermal stability, and ease of processing, it has become a material of choice for producing fibers, films, and membranes. Furthermore, the integration of nanomaterial additives has expanded PVA’s applications, enhancing its functionality and utility in various domains [15].

In this literature, it will be limited only to the published papers that deal with a TGA, device on PVA material. There are two groups. The first one studies the kinetics and mechanisms of the reaction, and this is what we focus more on, and the second group is used to allow for other studies such as the stability of PVA. But there is still room for comparison in the expected reactions from TGA, and derivative thermogravimetric analysis (DTG) curves.

Zhao et al. (2023) [16] aimed to determine the kinetics paramters and the reaction mechanism for PVA pyrolysis using TGA under inert nitrogen at different heating rates. They identified two reactions within the whole pyrolysis, and calcualted the activations for these reactions using different model free methods. They tried to figure out the convenient reaction model using CR and the masterplot. They concluded that F3/2 and F2 are the most approriate mechanism for the two reactions, but failed to give any clear justification for the overlapping between the two reactions.

Mittal et al. (2020) [17] used TGA of the PVA film to analyse the kinetic pyrolysis under a nitrogen gas from 303 to 1173 K, at four heating rates of 5, 10, 15, and 20 K min^−1^. They found commonly notice that the increase of heating rate from 5 to 20 K min^−1^, TG and DTG curves were extended towards higher temperatures without any changs in the total mass loss and pattern degradation. Out of four different heating rates, 10 °C min^−1^ will be presented widely by them. They showed for this heating rate only one single step weight loss of 98.72% within temperature range (473–873 K) for the degradation of PVA polymer backbone releasing organic volatiles. They performed FWO, KAS, FR, and modified CR methods to determine the activation energy for conversion range (0.1–0.9).

Wang et al. (2018) [18] studied the thermal properties of PVA using TGA by heating the sample from 50 to 600 °C with four different heating rates. Their TG showed a two-step reaction, one between 503 and 593 K, followed by another reaction between 593 and 773 K. They calculated the activation energy between 88 and 151 kJ mol^−1^ using the FWO method.

Thermal behaviors of the PVA polymer membrane were studied by TGA with temperature 30–750 °C under inert nitrogen at single heating rate 10 K min^−1^ [19]. TGA showed three clear degradations at 396 K (loss of water), 650 K (elimination of side-groups), and 707 K (decomposition and carbonization of macromolecular). Thermal behavior of another PVA membrane was implemented using TGA at heating rate 10 K min^−1^ up to temperature 1073 K [20]. Similar to the above paper (Radoor et al. (2024) [19]), three weight loss regions, 323–473 K (water evaporation); 473–873 K (polymer decomposition); and 600–680 °C (splitting polymer residues to organic hydrocarbons. Yan et al. (2020) [15], ran the TGA for the pyrolysis of PVA from 303 to 873 K at heating rate 10 K min^−1^. They found four weight loss stages during the process with the main decomposition process from 523 to 723 K. Reguieg et al. (2020) [21] conducted similar work to the above papers by running TGA from 303 to 1173 K under nitrogen at 10 K min^−1^. As in [19,20,21], PVA lost weights were in four main steps through TGA.

This study aims mainly to collect kinetics parameters for PVA pyrolysis from TGA by six model-free methods, since there are few papers covering the kinetics reaction. The mechanism of the reaction was investigated by two model-fittings. Moreover, thermodynamic parameters of the reaction have been calculated and confirmed that confirmed the reaction is endothermic.

## 2. Experimental Methods

### 2.1. PVA Material with TGA

For this study, the poly(vinyl alcohol) used was that of Industrial and Scientific grade 1788 L powder with the following characteristics: molecular weight of 74,800 kDa, 88% alcoholysis and 1700 polymerization degree. Heating rate variations (20, 30, and 40 K min^−1^) were studied using TGA (Mettler Toledo, Columbus, OH, USA) on samples (5–10 mg) heated from 298 to 873 K under a nitrogen atmosphere (40 mL/min flow rate). TGA data for weight loss of these PVA samples as well as temperature data for degradation were extracted from the TGA device and prepared for kinetics calculation.

### 2.2. Kinetic Equations Derivation

The derivation of PVA pyrolysis reaction will be based on the following Arrhenius equation:

(1) $$\frac{d\alpha }{dt}=A\exp\left(-\frac{E_{a}}{RT}\;\right)f\left(\alpha \right)$$

where all these symbols were found in [22,23,24].

*β* (°C/min) could be involved if there is a non-isotherm run as follows:

(2) $$\beta \frac{d\alpha }{dT}=A\exp\left(-\frac{E_{a}}{RT}\;\right)f\left(\alpha \right)$$

Equation (2) will be used to derive all six model-free methods by following different assumptions used for each method [22,23,24] (Table 1). The Criado method, as detailed in Table 2, leverages common solid-state thermal reaction mechanisms (*f*(*α*) and *g*(*α*)) to differentiate between theoretical predictions (left side of Equation (10)) and experimental observations (right side). This comparison, showcased in Table 3, ultimately helps determine the most accurate kinetic model for the given reaction.

### 2.3. Thermodynamic Parameters of PET Pyrolysis

The thermodynamic characteristics of PVA pyrolysis based on the calculated values of (*E_a_*, *A*_0_, and *T_p_*), can be determined by the three following equations:

(11) $$∆H=E_{a}-R\;T_{p}$$

(12) $$∆G=E_{a}+R\;T_{p}ln\left(\frac{k_{B}\;T_{p}}{h\;A}\right)\;$$

(13) $$∆S=\frac{∆H-∆G}{T_{p}}$$

All these symbols with the definition and numbers can be found in [24].

## 3. Results and Discussion

### 3.1. The TG Analysis

**Figure 1:**
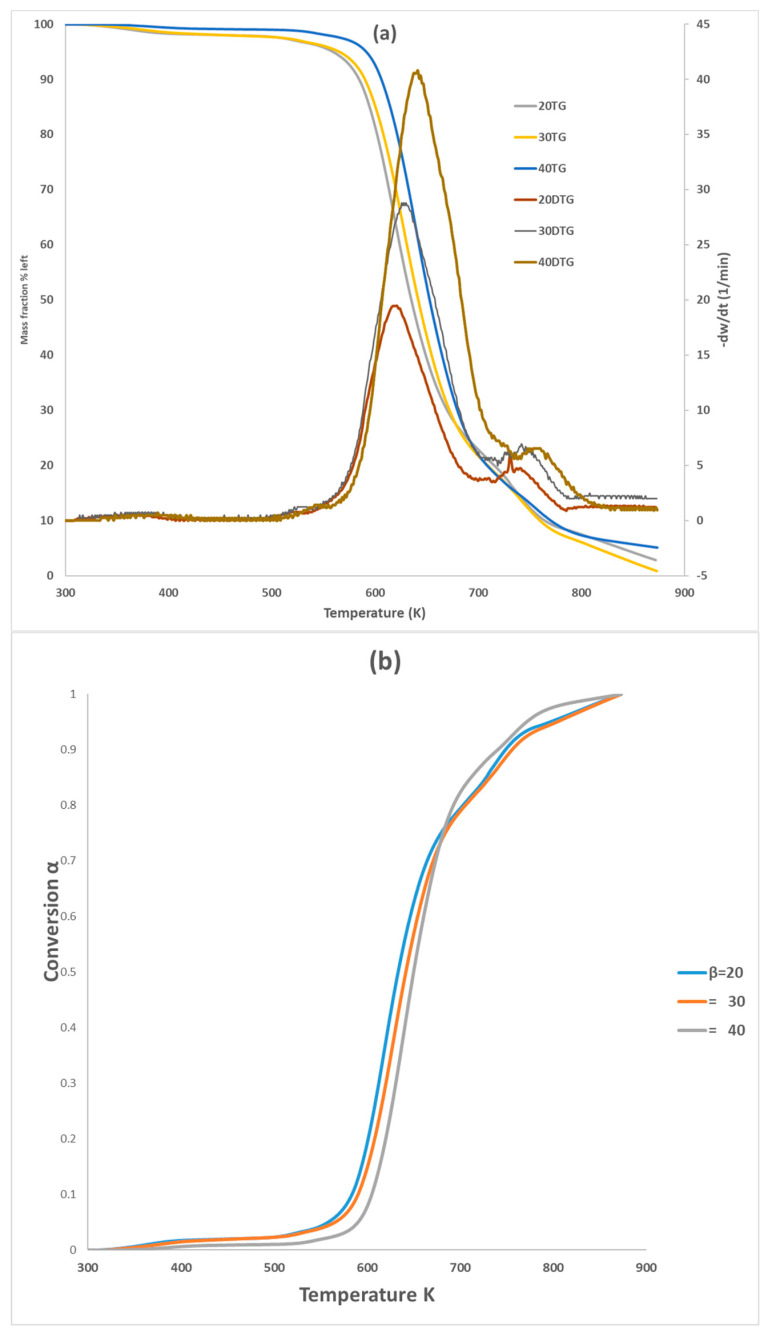
TG and DTG curves (**a**) and conversion (**b**) of PVA pyrolysis with three heating rates.

Figure 1 showcases the TG and DTG curves of PVA pyrolysis at heating rates of 20, 30, and 40 K min^−1^. The thermal decomposition behavior of PVA at different heating rates (20, 30, and 40 K min^−1^) is depicted in Figure 1 using TG and DTG curves. All three test samples were labeled with PVA20, PVA30, PVA40 labels throughout the paper to differentiate between different heating rates. The curves exhibit nearly identical behavior, but increasing the heating rate progressively shifts them to the right, particularly within the conversion range of 0.1 to 0.7. This suggests that a higher heating rate might influence the kinetics of PVA pyrolysis (Chowdhury et al. (2023) [22]). This phenomenon can be ascribed to the constraints imposed by heat transfer [26] and occurred with a higher heating rate, where the reactant will not have enough time to react. Figure 1b shows the pyrolysis of PVA occurred in two-step reactions; the main one at 550–750 K with about 80% weight loss (water elimination, chain scissions to produce acetaldehyde, saturated and unsaturated aldehydes, ketones, and some volatiles) and the second with 700–810 K (intermolecular cyclization to produce volatile gases, and Char). It can be noticed that by increasing the heating rate, the peak for DTG increases for both reactions. In this work, a temperature range of 550–810 K was selected to concentrate on pyrolysis behavior of PVA. Table 4 presented the pyrolysis characteristic temperature for the three tests. Wang et al. (2018) [18] also highlighted that the PVA pyrolysis can be considered as two steps; one between 230 and 320 °C for elimination reactions, followed between 320 and 500 °C for chain scission and cyclization reactions. For instance, Zhao et al. (2023) [16] elucidated that the TG and DTG curves have very similar behavior with two peak reactions, (550–660 K) and (700–800 K), if they are compared with our curves for the common heating rate 20 K min^−1^. The difference in the characteristic temperatures between the published papers could be attributed to the experimental conditions, sample source and size, operating pressure, and carrier gas flow rate. This can be explained that by increasing the heating rate, samples require a higher peak temperature to establish the same decomposition rate.

### 3.2. Model-Free Methods

**Figure 2:**
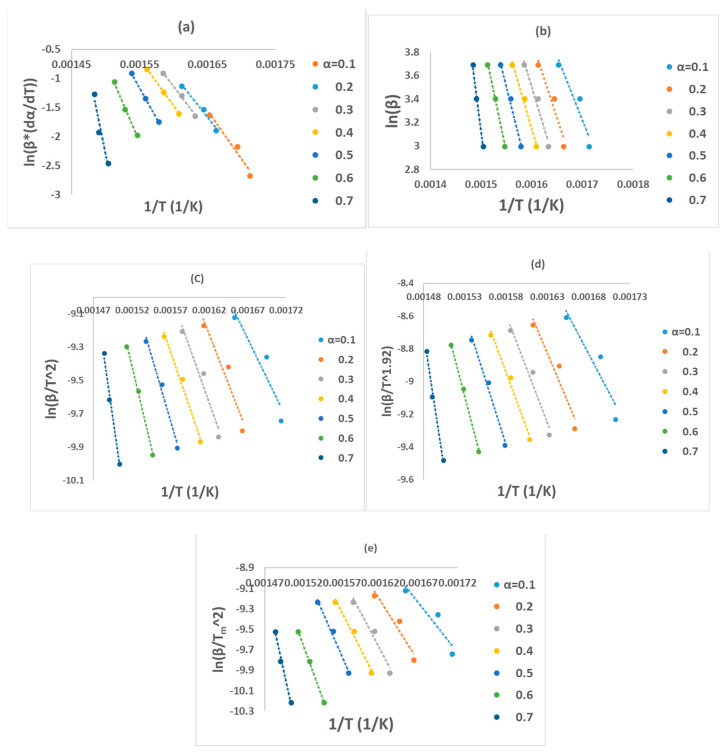
Regression lines of PVA pyrolysis by (**a**) FR, (**b**) FWO, (**c**) KAS, (**d**) STK, and (**e**) K models.

**Figure 3:**
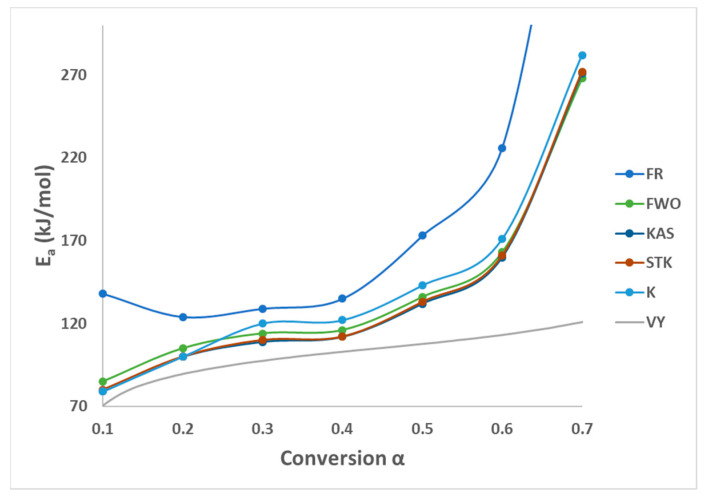
Activation energy by six methods of PVA pyrolysis.

All these six methods are the most effective and reliable to calculate the activation energy for non-isothermal, isoconversion, multiple heating rate. The difference between them is the assumptions that have been made for each method in deriving the final model. Activation energy (*E_a_*) represents the minimum energy barrier a reaction must overcome. Higher *E_a_* values result in decreased reaction rates, as fewer collisions possess the necessary energy. Equations (3)–(7) are applied by plotting ($\ln\left(\beta \frac{d\alpha }{dT}\right),\;\ln\left(\beta \right),\;\ln\left(\frac{\beta }{T^{2}}\right),\;ln\frac{\beta }{T^{1.92}}$, $ln(\frac{\beta }{T_{m}^{2}})\;)$ on *Y*-axis versus 1/T in the conversion range (0.1–0.7) as shown in Figure 2. Figure 1b shows that above 0.75 conversion, the normal trend “increasing the heating rate progressively shifts them to the right” has been disturbed. Therefore, only (0.1–0.7) conversions have been selected in this calculation since the model-free methods used more than one heating rate. Using different kinetic models, the activation energy was calculated from the slopes of fitted straight lines to the curves. The data for activation energy as a function of the conversion range (0.1–0.7) is presented in Figure 3 and Table 5. Kinetic calculation software (software version: 1) has been used to solve the Vyazovkin method (Drozin et al. (2020) [27]). This paper introduces the basic features of this software. Activation energy values for conversion range (0.8–0.9) have been neglected because the normal trend “shifting to the right as the heating rate increases” is not present, but still Figure 1a shows a very clear small reaction. Table 5 presented the activation energy values as a function of the conversion range (0.1–0.7) for six model-free models. The conversion range (0.1–0.7) for all methods (Figure 3) could be divided into two regions; one with (0.1–0.5) range, where the activation energy values almost constant; and the second one with higher than 0.5 conversion, and *E_a_* values that would slowly increase until conversion 0.7. Moreover, the average activation energy (280 kJ mol^−1^) at 0.7 conversion is the highest value compared with the rest of the conversion (0.1–0.6). This difference could be attributed to the beginning of the second reaction. Notably, the activation energies calculated by FWO, KAS, ST, and K methods exhibited the same trend across the conversion range, confirming the reliability of these values. However, consistent with previous studies, FR methods yielded slightly higher activation energy values compared to the first four methods. Mittal et al.’s (2020) [17] study revealed that the formation of thermally stable char above 0.6 conversion significantly impacted the pyrolysis of PVA, as evidenced by a tenfold increase in activation energy for the FR model (from 86.28 to 986.16 KJ mol^−1^) as conversion progressed from 0.1 to 0.9.

By checking Figure 2a–e, it has been noticed that while moving from 0.1 conversion to 0.7, the slope which is a function of the activation energy, are increasing, and this observation has been confirmed by changing the average activation energy values from 89 kJ mol^−1^ at conversion 0.1 to 280 kJ mol^−1^ at conversion 0.7 with average 145 kJ mol^−1^. This high value of activation energy value at high conversion is attributed to the beginning of the second reaction which has not been included in this calculation (Zhao et al. (2023) [16]). Table 6 presents activation energy from three published papers. Two out of these three considered only one reaction with slightly low activation energy value 122.5 kJ mol^−1^ by Wang et al. (2018) [18], and high activation energy value (average = 304.40 kJ mol^−1^) by Mittal et al. (2020) [17]. Mittal et al. (2020) [17] mentioned that the FR method is more accurate than other model-free methods when *E_a_* values change with conversion range based on previous papers. They showed the activation energy by FR method changed from 86.28 kJ mol^−1^ at 0.1 conversion to 986.16 kJ mol^−1^ at 0.9 conversion. This increase in *E_a_* value has been attributed to the formation of thermally stable char at a conversion greater than 0.6. The third paper considered two reactions, the first one with an average activation energy value of 136.50 kJ mol^−1^, while the second reaction has an average of 261.30 kJ mol^−1^ [16].

### 3.3. Model-Fitting Methods

In this section, the main task is to apply these 15 kinetic solid-state reaction models mentioned in Table and compare the results with the experimental one. To determine the most favorable reaction mechanisms for PVA pyrolysis, the CR model was employed. Linear regressions based on Equation (9) yielded values of *E_a_*, *lnA*_0_, and R^2^ for each test run and all proposed solid-state reaction mechanisms, which are presented in Table 3. Here, R^2^ represents the linear fitting degrees between the experimental data and the theoretical model for the two reactions zone for the three different heating rates. The collected kinetic parameters for two step reactions are presented in Table 7. Table 7 shows that the CR method with 15 equations of *g(α)* (F1–P4) is good fit with acceptable linear regression coefficient of R^2^ > 0.95. A big deviation in the value of *E_a_* was noticed within the range of 1–207 kJ min^−1^ for different mechanisms (F1–P4) of reaction.

**Figure 4:**
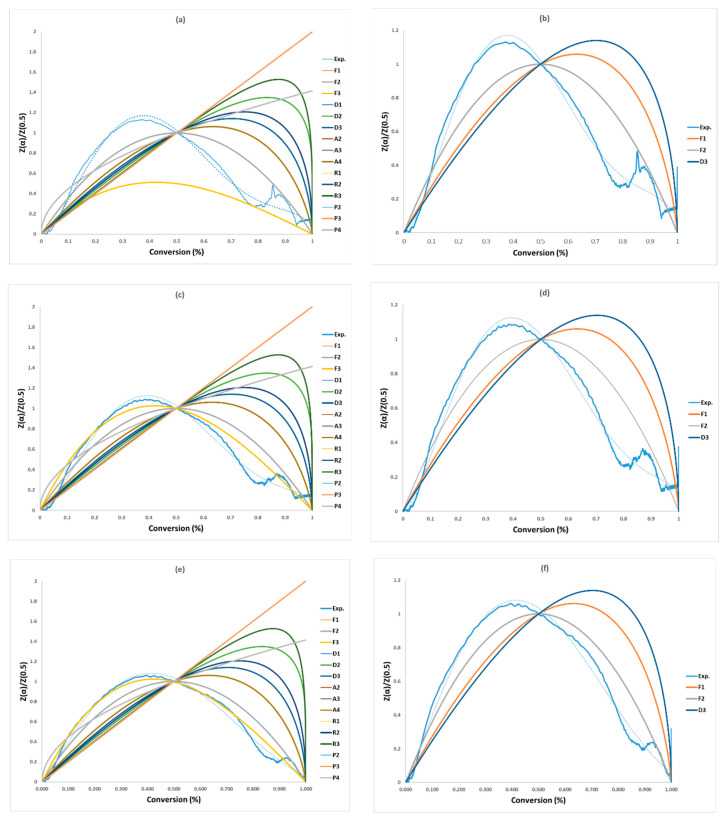
Masterplots of different kinetic models: (**a**,**b**) PVA20, (**c**,**d**) PVA30, (**e**,**f**) PVA40.

The non-linear masterplots method (Criado), presented in Figure 4 and Table 8, was used to check the precisions of the selected models by the CR method and identify the most appropriate activation energy value. Within the five model series (F, D, A, R, and P) of solid-state reactions, the F-series (F1–F2) and D-series (D3) are closest to the experimental data for the three heating rates, and among of them (F1, F2, and D3), F2 is the best model as shown in Figure 4b,d,f and Table 8. Therefore, F1, F2, and D3 were considered due to their agreement with the Criado plots in Figure 4b,d,f. Vyazovkin et al. (2020) [28] supports this finding when they highlighted that the decomposition reaction for solid material could be described by the F-series. These mechanisms are shown in Table 8 along with their activation energy (*E_a_*), pre-exponential factor (*lnA*_0_), and correlation coefficient (R^2^) for each of the three tests. Ultimately, the “*g(α)*-F2” mechanism was chosen and combined with the models FR, FWO, KAS, STK, and K in Table 9 to determine the pre-exponential factor. Zhao et al. (2023) [16] presented 19 solid-state reaction models (five kinds of solid-state models) for the first stage within conversion range of 0.02–0.7 and ended up with only “F-series” solid-state models, where they have the closest relationship with the experimental than the four solid models. Finally, out of these “F-series”, they concluded that the F3 model is considered as the best for all heating rates for the first reaction (conversion: 0.02–0.7) and F2 for the second reaction (conversion: 0.9–0.99). Vyazovkin et al. (2020) [28] supported this conclusion, suggesting that pyrolysis reactions for any solid material can be described. The findings lend support to this conclusion, indicating that pyrolysis reactions for all solid materials can be depicted.

**Figure 5:**
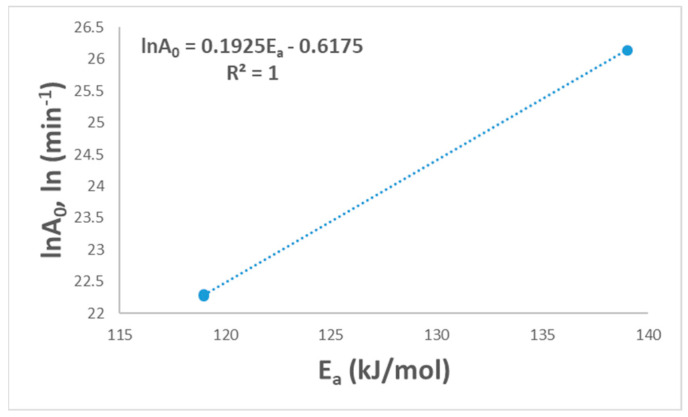
Linear fitted curve for the compensation effect.

The final selection mechanism could be applied to find the linearity between *lnA*_0_ and *E_a_*. Figure 5 shows the linear relationship with a (R^2^ = 1.0), and this shows the suitability of the suggested model for PVA pyrolysis. This relationship between *A*_0_ and *E_a_* is called the kinetic compensation effect [16].

### 3.4. Thermodynamic Parameters

To assess the energy feasibility of the pyrolysis process, which aims to produce energy (Chowdhury, 2023) [22], thermodynamic parameters were calculated alongside the kinetic ones. Table 10 presents the enthalpy change (∆H), Gibbs free energy change (∆G), and entropy change (∆S) for each heating rate. Positive values of ∆H (193.85, 193.76, and 193.68 kJ mol^−1^ for 20, 30, and 40 K min^−1^) indicate that the main reaction is endothermic, meaning it absorbs heat. This implies that external energy input is required to sustain the pyrolysis process. Again, positive values of ∆G confirm that the reaction is nonspontaneous and needs external heat to proceed with the reaction. Zhao et al. (2023) [16] showed that ∆H and ΔG for the first reaction (water elimination and random chain scission) is lower than the second reaction (intermolecular cyclization). These findings suggest that the residues remaining after PVA pyrolysis exhibit enhanced resistance to decomposition in comparison to the parent material. The data indicate that the pyrolytic conversion of PVA generates residues with a higher activation energy for subsequent decomposition, suggesting increased thermal stability. Usually, the change in entropy ∆S of a process was used to figure out the disorder of the system. From Table 10, ∆S has a value close to zero, and this means that the system is less disordered [22].

## 4. Conclusions

This study investigated the pyrolysis of PVA at different heating rates, revealing a two-stage process. While both stages are discussed, only the main one (550–750 K) was used for kinetic analysis. Six model-free methods were employed to determine the activation energy. To identify the most likely reaction mechanism, a combination of the Coats–Redfern and Criado’s master plot analysis was used. This approach determined a second-order reaction (F2) as the preferred mechanism, further confirmed by the compensation effect. The thermodynamic analysis indicated that PVA pyrolysis is an endothermic process.

## Figures and Tables

**Table 1:** Equations for model-free methods [24,25].

Method	Formula		Draw
FR	$\ln\left(\beta \frac{d\alpha }{dT}\right)=\ln\left[A_{0}f(\alpha \right)]-\frac{E_{a}}{RT}$	(3)	$\ln\left(\beta \frac{d\alpha }{dT}\right)\;vs.\;\frac{1}{T}$
FWO	$\ln\left(\beta \right)=ln\frac{A_{0}E_{a}}{Rg(\alpha )}-5.331-1.052\frac{E_{a}}{RT}$	(4)	$\ln\left(\beta \right)\;vs.\;\frac{1}{T}$
KAS	$\ln\left(\frac{\beta }{T^{2}}\right)=ln\frac{A_{0}R}{E_{a}\;g(\alpha )}-\frac{E_{a}}{RT}$	(5)	$\ln\left(\frac{\beta }{T^{2}}\right)\;vs.\;\frac{1}{T}$
STK	$ln\frac{\beta }{T^{1.92}}=ln\left(\frac{A_{0}E_{a}}{Rg(\alpha )}\right)-1.0008\frac{E_{a}}{RT}$	(6)	$ln\frac{\beta }{T^{1.92}}\;vs.\;\frac{1}{T}$
K	$ln(\frac{\beta }{T_{m}^{2}})=ln\left(\frac{A_{0}R}{E_{a}}\right)-\frac{E_{a}}{RT}$	(7)	$ln(\frac{\beta }{T_{m}^{2}})\;vs.\;\frac{1}{T}$
VY	$\Phi \left(E_{\alpha }\right)=\sum_{i=1}^{n}\sum_{j\neq i}^{n}\frac{J[E_{\alpha },\;T_{i}\left(t_{\alpha }\right)]}{J[E_{\alpha },\;T_{j}\left(t_{\alpha }\right)]}=0$	(8)	minimizing the function $\Phi \left(E_{\alpha }\right)$

**Table 2:** Equations for model-fitting methods [24].

Method	Formula	
CR	$ln\left[\frac{g(\alpha )}{T^{2}}\right]=ln\left[\frac{A_{0}R}{\beta E_{a}}\right]-\frac{E}{RT}$	(9)
Criado	$\frac{Z(\alpha )}{Z(0.5)}=\frac{f\left(\alpha \right)g(\alpha )}{f\left(0.5\right)g(0.5)}={\left(\frac{T_{\alpha }}{T_{0.5}}\right)}^{2}\;\frac{{\left(\frac{d\alpha }{dt}\right)}_{\alpha }}{{\left(\frac{d\alpha }{dt}\right)}_{0.5}}\;\;$	(10)

**Table 3:** Solid-state thermal reaction mechanism [24].

Model Series	Reaction Mechanism	Code	f(α)	g(α)
F	Reaction order models–1st order	F1	1 − *α*	−ln⁡(1−α)
	2nd order	F2	${\left(1-\alpha \right)}^{2}$	${\left(1-\alpha \right)}^{-1}-1$
	3rd order	F3	${\left(1-\alpha \right)}^{3}$	${\left[(1-\alpha \right)}^{-1}-1]/2$
D	Diffusion model–1 dimension	D1	$1/2\alpha^{-1}$	$\alpha^{2}$
	2 dimension	D2	${\left[-ln(1-\alpha )\right]}^{-1}$	$\left(1-\alpha \right)ln\left(1-\alpha \right)+\alpha$
	3 dimension	D3	$3/2{\left[1-{\left(1-\alpha \right)}^{1/3}\right]}^{-1}$	${\left[1-{\left(1-\alpha \right)}^{1/3}\right]}^{2}$
A	Nucleation models–2 dimension	A2	$2(1-\alpha ){\left[-\ln\left(1-\alpha \right)\right]}^{1/2}$	${\left[-\ln\left(1-\alpha \right)\right]}^{1/2}$
	3 dimension	A3	$3(1-\alpha ){\left[-\ln\left(1-\alpha \right)\right]}^{1/3}$	${\left[-\ln\left(1-\alpha \right)\right]}^{1/3}$
	4 dimension	A4	$4(1-\alpha ){\left[-\ln\left(1-\alpha \right)\right]}^{1/4}$	${\left[-\ln\left(1-\alpha \right)\right]}^{1/4}$
R	Geometrical contraction models–One dimension	R1	1	α
	- sphere	R2	$2{\left(1-\alpha \right)}^{1/2}$	$1-{\left(1-\alpha \right)}^{1/2}$
	- cylinder	R3	$3{\left(1-\alpha \right)}^{1/3}$	$1-{\left(1-\alpha \right)}^{1/3}$
P	Nucleation models–2-Power law	P2	$2\alpha^{1/2}$	$\alpha^{1/2}$
	3-Power law	P3	$3\alpha^{2/3}$	$\alpha^{1/3}$
	4-Power law	P4	$4\alpha^{3/4}$	$\alpha^{1/4}$

**Table 4:** Characteristic temperatures of PVA pyrolysis.

Test No.	Heating Rate (K min^−1^)	Symbol	Step 1 Reaction			Step 2 Reaction		
			On-Set Temp. (K)	Peak Temp. (K)	Final Temp. (K)	On-Set Temp. (K)	Peak Temp. (K)	FinalTemp. (K)
1	20	PVA20	550	620	700	700	740	790
2	30	PVA30	560	630	720	720	750	800
3	40	PVA40	570	640	730	730	760	810

**Table 5:** Activation energy values obtained by six model-free methods.

Conversion	FR		FWO		KAS		STK		K		VY		Average	
	E (kJ/mol)	R^2^	E (kJ/mol)	R^2^	E (kJ/mol)	R^2^	E (kJ/mol)	R^2^	E (kJ/mol)	R^2^	E (kJ/mol)	R^2^	E (kJ/mol)	R^2^
0.1	138	0.9623	85	0.9022	79	0.8791	80	0.8801	79	0.8791	71	NA *	89	0.90056
0.2	124	0.9856	105	0.9387	100	0.9264	100	0.9269	100	0.9264	90	NA *	103	0.9408
0.3	129	0.9945	114	0.9623	109	0.9551	110	0.9554	120	0.9623	97	NA *	113	0.96592
0.4	135	1	116	0.9863	112	0.9835	112	0.9836	122	0.9863	130	NA *	121	0.98794
0.5	173	0.9998	136	0.9886	132	0.9866	133	0.9867	143	0.9886	108	NA *	138	0.99006
0.6	226	0.9949	163	0.9978	160	0.9975	161	0.9975	171	0.9978	113	NA *	166	0.9971
0.7	468	0.9278	268	0.9867	271	0.9856	272	0.9856	282	0.9867	121	NA *	280	0.97448
Average	199	0.9807	148	0.9961	138	0.9591	138	0.9594	145	0.9610	104	NA *	145	0.97126

NA *: Kinetic calculation software does not provide this information.

**Table 6:** Activation energies from different published papers.

	1st Stage		2nd Stage	
References	E (kJ mol^−1^)	Method	E (kJ mol^−1^)	Method
Zhao et al. (2023) [16]	135.97	FWO	269.34	FWO
	133.78	KAS	271.16	KAS
	142.20	FR	234.33	FR
	134.05	AIC	270.38	AIC
Mittal et al. (2020) [17]	298.73	FWO		
	304.55	KAS		
	309.67	FR		
	304.64	Modified CR		
Wang et al. (2018) [18]	122.5	FWO		

**Table 7:** Kinetic parameters obtained by CR model.

Reaction Mechanism 1 Step Reaction	Code	Test 1 PVA20			Test 2 PVA30			Test 3 PVA40		
		*E_a_* (kJ/mol)	*Ln* (*A*_0_)	R^2^	*E_a_* (kJ/mol)	*Ln* (*A*_0_)	R^2^	*E_a_* (kJ/mol)	*Ln* (*A*_0_)	R^2^
Reaction order models–First order	F1	91	16.28	0.9912	92	16.67	0.9878	107	19.66	0.9868
Reaction order models–Second order	F2	119	22.3	0.9969	119	22.28	0.9971	139	26.14	0.9974
Reaction order models–Third order	F3	152	29.21	0.9993	150	28.71	0.9998	176	33.63	1
Diffusion models–One dimension	D1	145	25.96	0.9829	151	27.07	0.9729	174	31.31	0.9682
Diffusion models–Two dimension	D2	159	28.27	0.9864	164	29.16	0.979	189	33.78	0.9757
Diffusion models–Three dimension	D3	175	30.17	0.9897	179	30.81	0.9848	207	35.89	0.9828
Nucleation models–Two dimension	A2	40	16.92	0.9886	41	17.37	0.9842	195	33.48	0.9783
Nucleation models–Three-dimension	A3	23	19.51	0.9848	24	19.98	0.9788	48	16.56	0.9835
Nucleation models–Fourth dimension	A4	15	20.65	0.9789	15	21.08	0.9704	29	19.63	0.9788
Geometrical contraction models–One dimension phase boundary	R1	67	12.83	0.98	70	12.95	0.9685	19	20.99	0.972
Geometrical contraction models–Contracting sphere	R2	78	12.9	0.9865	81	13.5	0.9796	82	14.26	0.9638
Geometrical contraction models–Contracting cylinder	R3	82	13.37	0.9882	84	13.89	0.9827	94	16.14	0.9771
Nucleation models–Power law	P2	9	21.34	0.931	30	19.29	0.9561	98	16.64	0.9807
Nucleation models–Power law	P3	15	20.69	0.9578	16	21.06	0.9355	35	18.81	0.952
Nucleation models–Power law	P4	28	18.97	0.9719	10	21.79	0.8981	28	19.66	0.9994
**Reaction mechanism 2 step reaction**	**Code**	**Test 1 PVA20**			**Test 2 PVA30**			**Test 3 PVA40**		
		***E_a_* (kJ/mol)**	***Ln* (*A*_0_)**	**R^2^**	***E_a_* (kJ/mol)**	***Ln* (*A*_0_)**	**R^2^**	***E_a_* (kJ/mol)**	***Ln* (*A*_0_)**	**R^2^**
Reaction order models–First order	F1	29	18.85	0.9993	27	19.62	1	25	19.98	0.9933
Reaction order models–Second order	F2	92	16.01	0.9994	85	15.1	0.9996	90	16.52	0.9932
Reaction order models–Third order	F3	176	31.83	0.9991	163	29.86	0.9994	176	32.92	0.9932
Diffusion models–One dimension	D1	10	21.9	0.9795	9	22.39	0.9936	5	22.77	0.9961
Diffusion models–Two dimension	D2	22	21	0.994	20	21.6	0.9986	15	23.14	0.9961
Diffusion models–Three dimension	D3	44	19	0.9986	41	19.88	0.9998	36	20.68	0.9963
Nucleation models–Two dimension	A2	8	22.58	0.9977	7	21.82	0.9998	6	22.03	0.9754
Nucleation models–Three-dimension	A3	1	20.5	0.9654	1	21.06	0.991	NA	NA	NA
Nucleation models–Fourth dimension	A4	NA	NA	NA	NA	NA	NA	NA	NA	NA
Geometrical contraction models–One dimension phase boundary	R1	NA	NA	NA	NA	NA	NA	NA	NA	NA
Geometrical contraction models–Contracting sphere	R2	10	21.94	0.9931	9	22.56	0.9986	7	22.96	0.9883
Geometrical contraction models–Contracting cylinder	R3	16	21.89	0.9971	14	22.44	0.9996	12	22.89	0.9918
Nucleation models–Power law	P2	NA	NA	NA	NA	NA	NA	NA	NA	NA
Nucleation models–Power law	P3	NA	NA	NA	NA	NA	NA	NA	NA	NA
Nucleation models–Power law	P4	NA	NA	NA	NA	NA	NA	NA	NA	NA

**Table 8:** Activation energy of (CR and Criado).

Test No.	*E_a_* (kJ/mol)	*Ln* (*A*_0_)	R^2^	Reaction Mechanism
1	119	22.3	0.9969	Reaction order models-Second order (F2)
2	119	22.28	0.9971	Reaction order models-Second order (F2)
3	139	26.14	0.9974	Reaction order models-Second order (F2)

**Table 9:** Pre-exponential factor values obtained by isoconversional models.

Conversion		*ln* [*A*_0_ (min^−1^)]				
	FR	FWO	KAS	STK	K	Average
0.1	26.06	15.37	13.66	14.26	15.85	36.88
0.2	23.32	19.52	18.19	18.79	19.58	26.40
0.3	24.43	21.48	20.29	20.89	23.18	25.48
0.4	25.52	22.07	20.90	21.49	23.36	26.08
0.5	32.43	25.78	24.89	25.50	26.98	27.40
0.6	41.86	30.70	30.16	30.76	31.57	36.88
0.7	84.54	49.87	50.30	50.90	51.29	26.40
Average	17.04	19.88	22.05	22.67	27.12	28.45

**Table 10:** Thermodynamic parameters.

Heating Rates (K/min)	20	30	40
Kinetic Parameters			
*E_a_* (kJ/mol)	199		
*A* (min^−1^)	2.30 × 10^15^		
*T_p_* (K)	620	630	640
Thermodynamic Parameters			
∆*H* (kJ/mol)	193.85	193.76	193.68
∆G (kJ/mol)	172.30	171.94	171.60
∆*S* (kJ/mol.K)	0.034767	0.034634	0.034503
Potential Energy Barrier			
*E_a_* − ∆*H* (kJ/mol) *	5.15	5.32	5.24

* Based on the mean values of Δ*H*.

## Data Availability

Data are contained within the article.
